# A novel murine model of multi-day moderate ethanol exposure reveals increased intestinal dysfunction and liver inflammation with age

**DOI:** 10.1186/s12979-021-00247-8

**Published:** 2021-09-23

**Authors:** Rachel H McMahan, Kevin M Najarro, Juliet E Mullen, Madison T Paul, David J Orlicky, Holly J Hulsebus, Elizabeth J Kovacs

**Affiliations:** 1 Department of Surgery, Division of GI, Trauma and Endocrine Surgery, and Alcohol Research Program, University of Colorado Denver, Anschutz Medical Campus, 12700 East 19th Ave, RC2, Mail Stop #8620, CO 80045 Aurora, USA; 2 GI and Liver Innate Immune Program, University of Colorado Denver, Anschutz Medical Campus, Aurora, CO 80045 USA; 3 Department of Pathology, University of Colorado Denver, Anschutz Medical Campus, Aurora, CO 80045 USA; 4 Department of Immunology and Microbiology, University of Colorado Denver, Anschutz Medical Campus, Aurora, CO 80045 USA

**Keywords:** Alcohol, Aging, Gut barrier, Liver, Antimicrobial peptide

## Abstract

**Background:**

There are currently > 600 million people over the age of 65 globally and this number is expected to double by the year 2050. Alcohol use among this population is on the rise, which is concerning as aging is associated with increased risk for a number of chronic illnesses. As most studies investigating the effects of alcohol have focused on young/middle-aged populations, there is a dearth of information regarding the consequences of alcohol use in older consumers. In addition, most murine ethanol models have concentrated on exposure to very high levels of ethanol, while the vast majority of elderly drinkers do not consume alcohol in excess; instead, they drink on average 2 alcoholic beverages a day, 3–4 days a week.

**Methods:**

We designed a murine model of aging and moderate ethanol consumption to determine if the deleterious effects of alcohol on the gut-liver axis are exacerbated in aged, relative to younger, animals. Aged and young mice were exposed to a multi-day moderate exposure ethanol regimen for 4 weeks and changes in gut permeability along with intestinal tight junction protein and antimicrobial peptide gene expression were measured. In addition, hepatic inflammation was assessed by histological analysis, inflammatory gene expression and flow cytometric analysis of inflammatory infiltrate.

**Results:**

Our results reveal that in aged, but not young mice, moderate ethanol exposure yielded significantly worsened intestinal permeability, including increased bacterial translocation from the gut, elevated serum iFABP and leakage of FITC-dextran from the gut. Interestingly, moderate ethanol exposure in young animals led to gut protective transcriptional changes in the ileum while this protective response was blunted in aged mice. Finally, moderate ethanol exposure in aged mice also resulted in marked inflammatory changes in the liver.

**Conclusions:**

These results demonstrate that aged mice are more susceptible to ethanol-induced gut barrier dysfunction and liver inflammation, even at moderate doses of ethanol. This increased vulnerability to ethanol’s gastrointestinal effects has important implications for alcohol use in the aging population. Future studies will explore whether improving intestinal barrier function can reverse these age-related changes.

## Background

According to the World Health Organization, 8.5 % of the global population is currently > 65 years old and, over the next 40 years, older adults will outnumber children worldwide [1]. This is occurring alongside a simultaneous increase in the prevalence of alcohol use among older adults, with a 22 % increase from 2002 to 2014 [2]. Of the older adults who drink alcohol, approximately 90 % do not “binge drink,” which is defined as 4–5 drinks in a two hour period [3], but instead consume an average of 2 alcoholic drinks per day, 3–4 days a week [4–6]. While it is known that the elderly population is at increased risk for a number of diseases, there is a scarcity of literature regarding the effects of alcohol consumption in the aged, especially at the more moderate levels reflective of the alcohol consumption pattern for the majority of older drinkers.

Consumption of excessive amounts of alcohol over long periods of time can have wide-ranging effects on multiple organ systems including the gastrointestinal tract (reviewed in [7]). In the healthy gut, the intestinal barrier prevents the escape of microbes from the lumen while still allowing transport of dietary nutrients. This barrier is upheld, in part, by tight junction proteins that are present near the apical surface of intestinal epithelial cells, such as zonulin (ZO-1), claudins, and occludins (reviewed in [8]). In addition, there are chemical barriers generated by antimicrobial peptides (AMPs) which reside in the mucus layer on the luminal surface of the gut and are produced by both epithelial cells and immune cells [9]. One subset of AMPs, regenerating islet-derived proteins (Regs), are C-type lectin proteins that are upregulated in response to damage within the GI-tract. The Reg3 family members, Reg3γ and Reg3β, are highly expressed in the intestine. They play a protective role in the injured intestine, improving cell growth and survival [10] along with promoting barrier function by preventing the microbiota in the lumen of the intestine from invading intestinal epithelial cells [11] .

A number of murine studies have shown that chronic exposure to very high levels of ethanol can lead to disruption of the intestinal barrier, which, in turn, promotes a range of pathologies both inside and outside the gastrointestinal tract [12–14]. Ethanol and its metabolites have direct toxic effects on epithelial cells [15]. In addition, ethanol can induce altered expression of tight junction proteins [16] and AMPs [14, 17]. This disruption of both the physical and chemical components of the intestinal barrier leads to increased translocation of bacteria and bacterial products, known as microbial associated molecular patterns (MAMPs), to mesenteric lymph nodes, [14] and the liver [12]. In the liver, MAMPs can bind to pattern recognition receptors on resident cells and trigger hepatic inflammation [12, 18, 19].

While there has been extensive research investigating the effects of ethanol on multiple organ systems in young rodents and other mammalian models, there is a lack of research regarding its effect in advanced age. There is evidence of impaired intestinal function in aged mice and primates [20, 21], and the increase in gut-related disorders in the aged population could reflect reduced intestinal function in the elderly [22]. Weakened intestinal barrier function with advanced age would also have important implications for elderly individuals who consume alcohol, as alcohol-induced disruptions in the gut-liver axis contributes to its harmful effects. Therefore, to begin to elucidate the effects of ethanol exposure on the aging gastrointestinal system, we have established a clinically relevant murine model of ethanol consumption and advanced age. This model utilizes a more moderate ethanol exposure pattern than previous studies conducted in young adult mice [23–26], to better mimic the patterns and levels of alcohol exposure most often seen in the elderly [4–6]. Herein, we show that even moderate levels of ethanol exposure markedly impair intestinal integrity and induce hepatic inflammation in aged mice in response to ethanol. These findings have important implications for alcohol use in the aging population and validate this as a useful model for further study of moderate alcohol use in aged mice.

## Methods

### Mice

BALB/cBy mice were obtained from the National Institute on Aging (NIA) colony (Charles River Laboratories, Wilmington, MA). Mice were housed at the University of Colorado Anschutz Medical Campus vivarium for a minimum of 2 weeks prior to any experimentation. All animal experiments were performed humanely under a protocol approved by the Animal Care and Use Committee of the University of Colorado Anschutz Medical Campus (Protocol # 000587). Young mice were 4–5 months of age (similar to humans 25–30 years) and aged mice were 21–22 months of age (similar to humans > 65 years) [27]. Young and aged female mice were subjected to a multi-day ethanol exposure model in which mice were gavaged with ethanol (1.5 g/kg and 1.25 g/kg, respectively) on 3 consecutive days a week for 4 weeks (3 days on ethanol, 4 days off ethanol). As the rate of ethanol metabolism differs between young and older subjects [28], we adjusted ethanol dosages to elevate blood ethanol levels in both young and aged mice to an equivalent of ~ 80 mg/dL at 30 min post-gavage. Mice in the vehicle group were gavaged with a calorically equivalent dose of maltose dextran. A small amount of blood was collected 30 min after oral gavage of ethanol and blood alcohol content (BAC) was measured using the Ethanol Colormetric/Fluorometric Assay Kit from BioVision. The protocol was executed according to the manufacturer’s instructions using a 1:50 serum dilution. Sample absorbency at 570 nm was measured using a Thermo Scientific MultiSkan GO plate reader. One hour after the final ethanol gavage, mice were humanely euthanized using CO_2_ inhalation and blood and organs were collected for analysis.

### Histology

Liver and ileum were removed and fixed overnight in 10 % formalin before long-term storage in 70 % ethanol. Liver sections were stained with hematoxylin and eosin (H&E) and scored for injury in a blinded fashion as previously described [29, 30]. Briefly, lobular inflammation was scored by assessment of the number of inflammatory foci within a 200x field; No foci (0), 1–2 foci per 200× field (1), 3–4 foci per 200× field (2), > 4 foci per 200× field (3). Sinusoidal dilation was defined as having a diameter ≥ 3 red blood cells.

### Intestinal Epithelial Cell (IEC) isolation

The protocol for isolation of IEC was adapted from a previously described method [31]. Briefly, ileum tissue was harvested, flushed with 10 ml ice-cold phosphate-buffered saline (PBS) and excess fat and mesentery was removed. The tissue was cut longitudinally, washed and incubated in ice cold PBS with 30 mM EDTA and 1.5 mM DTT for 20 min, followed by incubation at 37 °C for 10 min in PBS with 30 mM EDTA. Epithelium was released from the basement membrane by shaking, and dissociated cells were disrupted in buffer RLT (Qiagen) and homogenized with a QiaShredder (Qiagen) before storing at -80 °C.

### Quantification of Mesenteric Lymph Node (MLN) bacteria

Bacterial translocation to the MLN was assessed as previously described [32]. Briefly, mice were euthanized one hour after the final ethanol dose and MLN were aseptically isolated and washed in 1 ml of sterile PBS. MLN were mechanically disrupted by gently dissociating between sterile frosted glass slides. 100 µl of the homogenate was plated on tryptic soy agar plates with 5 % sheep’s blood (Remel) and incubated at 37 °C, 5 % CO_2_. Bacterial colony-forming units (CFU) were counted at 24 h.

### Intestinal permeability assays

Intestinal permeability to fluorescein isothiocynate (FITC)-conjugated dextran (FITC-dextran, 4kD) was measured using a previously described protocol [33]. Briefly, three hours prior to euthanasia, mice were gavaged with FITC-Dextran (600 mg/kg of body weight) simultaneously with the final dose of ethanol. Serum was collected and 100 µl was added to 96 wells black walled, clear bottom plates along with a standard dilution of FITC-dextran. **S**amples were analyzed with a fluorescence spectrophotometer (520nm) using a Promega GloMax + plate reader. For quantification of intestinal fatty acid binding protein (iFABP) mouse serum was collected 1 h after the final ethanol gavage and iFABP concentrations were assessed by ELISA using a commercially available mouse iFABP ELISA Kit from MyBioSource. Samples were run in duplicate according to manufacturer’s instructions and absorbency at 450nm was measured using a Thermo Scientific MultiSkan GO plate reader.

### Flow cytometric analysis of liver non-parenchymal cells

Liver non-parenchymal cells were isolated and stained as previously described [34]. Cells were stained with fluorophore-conjugated antibodies directed against the following surface antigens: CD45 (30-F11; Biolegend), Ly6G (1A8; ThermoFisher (eBioscience)), CD11b (M1/70; ThermoFisher (eBioscience)) and F4/80 (BM8; ThermoFisher (eBioscience)). Flow cytometry was performed using a BD LSRII instrument and data were analyzed with Flow Jo V10 software.

### Quantitative real-time PCR

Liver and ileal tissue were harvested and stored as previously described [32, 34]. RNA isolation and quantitative RT-PCR was performed as previously described [35]. Briefly, RNA was isolated using the RNeasy Mini kit and converted to cDNA using the QuantiTect RT kit (both from Qiagen) following standard protocols. Real-time quantitative PCR was performed on the QuantStudio 3 Real-Time PCR System (Thermo Fisher) using TaqMan probes and reagents: *Tjp1* (Mm01320638_m1), *Ocln* (Mm00500912_m1), *Cldn1* (Mm01342184_m1), *Reg3g* (Mm00441127-m1), *Reg3b* (Mm00440616_g1), *Tnf* (Mm00443258_m1), *Il1b* (Mm00434228_m1), *Ccl2* (Mm00441242_m1), *Cxcl2* (Mm00436450_m) (ThermoFisher). Results were analyzed using the ΔΔCt algorithm [36]. Hydroxymethyl-bilane synthase (*Hmbs*) [37] (Mm01143545_m1) and eukaryotic 18 s rRNA (*18 s*) (4319413E) (ThermoFisher) were used as the endogenous control for liver and ileum, respectively.

### Statistical analysis

A two-way ANOVA with Tukey’s multiple comparisons statistical test was used to compare data between treatment groups. Prism 7.03 statistical analysis software (GraphPad) was used to perform the analysis, and a *p*-value of 0.05 or less was considered significant.

## Results

### Moderate ethanol exposure results in intestinal barrier dysfunction in aged but not young mice

**Fig. 1:**
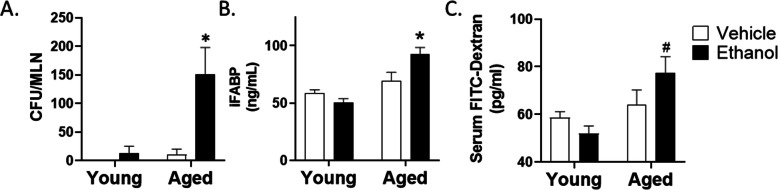
Moderate ethanol exposure induces increased bacteria within the mesenteric lymph node (MLN) and impaired barrier function in the intestine of aged, but not young mice. **A** Colony forming units (CFU) from MLNs isolated from young and aged mice following 4 weeks of ethanol or vehicle treatment. *n* = 4 mice per group. **B** Intestinal fatty acid binding protein (iFABP) levels in serum, measured by ELISA. **C** Concentration of 4 kDa FITC-dextran in serum of young and aged mice. *n* = 4–8 mice per group. Data are shown as mean values ± SEM. *p < 0.05 from all other groups, #*p* < 0.05 from young groups by two-way ANOVA with Tukey’s multiple comparisons test

To begin to evaluate whether the gastrointestinal effects of ethanol exposure are more dramatic in aged mice, we exposed aged and young mice to a multi-day moderate ethanol exposure regimen and examined a number of intestinal barrier parameters. Vehicle treatment of young mice had no demonstrable effects on bacterial translocation from the intestine as determined by a lack of bacterial colony growth from isolated MLNs (Fig. 1A). While young mice given ethanol and aged mice given vehicle had a small, non-significant amount of bacterial growth in the MLN, aged mice given moderate ethanol had substantial bacteria growth from the MLN, with a 12-fold and-15 fold increase compared to young ethanol and aged vehicle groups, respectively (*p* < 0.001). The existence of bacteria within the lymph node can be influenced by both increased epithelial permeability and impaired immunity at the mucosal surface [38]. Therefore, we examined other markers of intestinal permeability, including circulating levels of iFABP and leakage of gavaged 4kD FITC-dextran into the serum (Fig. 1B and C). Exposure of young mice to these moderate levels of ethanol had no effect on serum iFABP concentrations or serum FITC dextran levels, demonstrating that, at these concentrations, ethanol is not decreasing barrier function in young mice. In addition, these parameters were not significantly altered in our vehicle treated aged mice. Conversely, aged mice given ethanol had significantly higher levels of serum iFABP compared to young mice given ethanol (50.2 ± 3.5 vs. 92.8 ± 5.6 ng/ml; *p* < 0.005) (Fig. 1B), suggesting heightened epithelial cell damage in this treatment group. We also observed a significant 1.5-fold increase in serum FITC-dextran levels in aged mice given ethanol compared to young ethanol group (*p* < 0.01) (Fig. 1C). Taken together, these data support the hypothesis that ethanol consumption in age leads to a breakdown of the integrity of the intestinal epithelial barrier at ethanol levels that have no detrimental effects in young animals.

### Impaired intestinal responses in aged mice

**Fig. 2:**
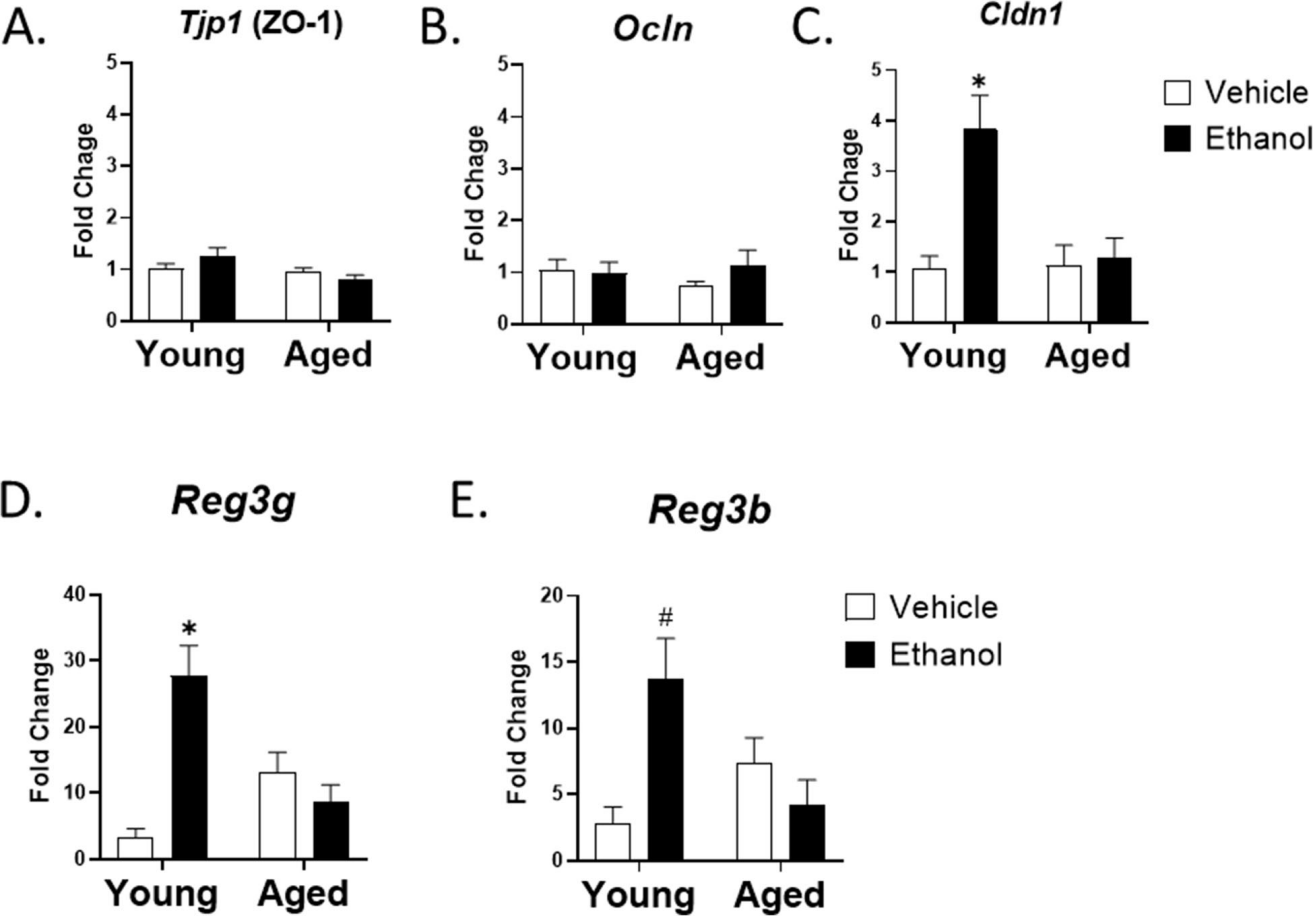
Moderate ethanol fails to induce ileal *Cldn1* and *Reg3* gene expression in aged mice. Quantitative RT-PCR of tight junction (**A-C**, *n* = 4**)** and AMP (**D-E**, *n* = 6–10) gene expression in intestinal epithelial cells (IECs) isolated from young and aged mice given vehicle or ethanol. *18 s* was used as the endogenous control. Data are shown as mean fold change ± SEM relative to young vehicle group. *p < 0.05 from all other groups, #*p* < 0.05 from young vehicle and aged ethanol by two-way ANOVA with Tukey’s multiple comparisons test

To explore possible explanations for the increased intestinal permeability with age in our model, we examined H&E stained ileum from the four treatment groups. Histological analysis of ileum showed no obvious changes in villus morphology or other histology (data not shown), suggesting there were no gross abnormalities in the intestinal epithelial cell barrier architecture between young and aged mice given vehicle or ethanol. The intestinal barrier is maintained, in part, by tight junction proteins expressed in intestinal epithelial cells (IEC). These include both cytosolic proteins (zona occludin-1 (ZO-1)) and membrane proteins (occludin and claudins) (reviewed in [8]). It has also been shown that high levels of ethanol can decrease levels of the tight junction proteins occludin and ZO-1 [13, 39, 40] however, we saw no changes in expression of ZO-1 (*Tjp1*) and Occludin (*Ocln*) in IEC isolated from the ileum of young and aged mice given vehicle or ethanol (Fig. 2A and B). In addition, immunofluorescence staining for ZO-1 and occludin showed a normal staining pattern in all treatment groups (data not shown). In contrast, expression of Claudin-1 (*Clnd1*), critical for maintaining the “tightness” of epithelial cell tight junctions [41], was upregulated 1.5 fold (*p* < 0.005) in IEC from young mice exposed to moderate ethanol compared to age-matched vehicle controls (Fig. 2C), likely as a compensatory mechanism. Interestingly, the ethanol-induced increase in *Clnd1* was absent in aged mice, suggesting an impaired intestinal response to ethanol-induced upregulation of this protein. Expression of other claudins did not differ between intestinal epithelial cells isolated from the ileum of the four treatment groups (data not shown).

In addition to tight junction regulated cell-cell connections, the intestinal epithelial barrier is also regulated by expression of the AMPs Reg3γ and Reg3β. In our model of moderate ethanol exposure, we observed a 9-fold (*p* < 0.0001) and 5-fold (*p* < 0.005) increase in ileal expression of *Reg3g* and *Reg3b* in young mice exposed to ethanol when compared to young mice given vehicle (Fig. 2D and E). In contrast, there was no ethanol-mediated rise in *Reg3g* or *Reg3b* in the ileum of aged mice. This is a similar pattern to that observed with *Cldn1*, further suggesting an impaired compensatory response to moderate ethanol in aged mice. Interestingly, there was also a baseline 4-fold elevation of *Reg3g* in the ileum of aged vehicle treated mice relative to the young vehicle group, indicating a possible baseline upregulation of AMPs with age.

### Moderate ethanol levels induce inflammatory changes in the aged liver

**Fig. 3:**
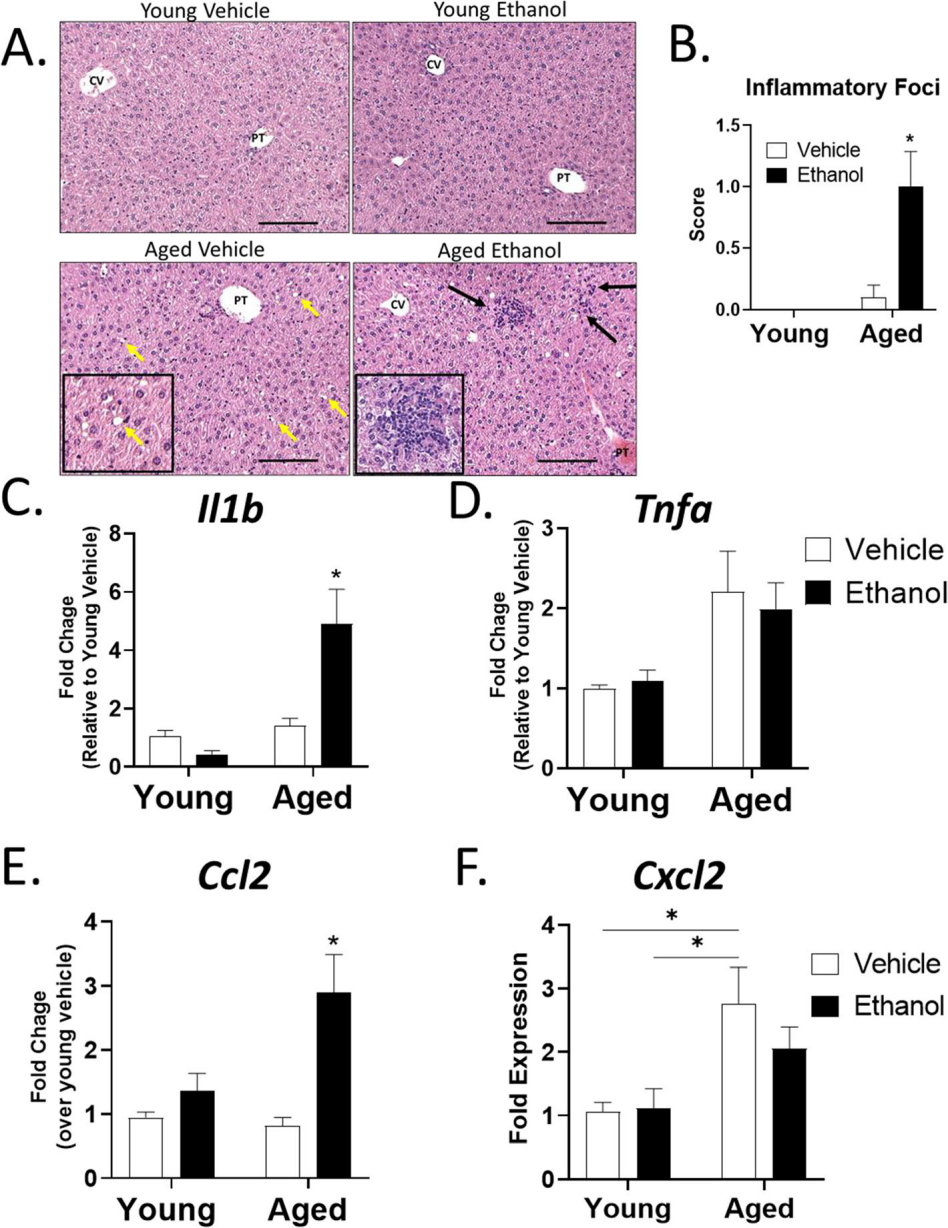
Moderate ethanol exposure in aged mice leads to increased hepatic inflammation and pro-inflammatory gene expression.** A **Representative H&E staining (200x) of livers from young and aged mice given vehicle or ethanol showing dilated sinusoids (yellow arrows and inset representing magnification of the upper right yellow arrow) in aged mice and increased foci of inflammatory cells (black arrows and inset showing magnification of the upper left black arrow) in aged mice given ethanol. Scale bars, 100 µM. PT = portal tract, CV = central vein. **B** Quantitative scoring of average number of inflammatory foci per 200x field for each group (0 = no foci, 1 = 1–2 foci, 3 = 3–4 foci). *n* = 4 per group and is representative of 3 individual experiments. **C-F.** Quantitative RT-PCR of pro-inflammatory and chemokine gene expression from the livers of mice with *Hmbs* as the endogenous control. Data are shown as mean fold change ± SEM relative to the young vehicle group unless otherwise indicated. *n* = 6 per group; *p < 0.05 from all other groups, #*p* < 0.05 from young groups by two-way ANOVA with Tukey’s multiple comparisons test

Inflammation within the liver can originate from the breakdown of the intestinal barrier and leakage of bacterial derived MAMPs into the liver (reviewed in [42]). In addition, liver injury induced by consumption of high amounts of ethanol is mediated, at least in part, by the breakdown of the intestinal barrier [17, 43, 44]. Therefore, we next examined liver inflammation in young and aged mice given moderate ethanol or vehicle. Histological analysis of H&E stained livers showed no changes in the liver of young mice exposed to the multi-day moderate ethanol protocol (Fig. 3A). In addition, livers from vehicle treated aged mice also showed no signs of hepatic inflammation, although heightened sinusoidal dilation was observed in these livers. In contrast, the presence of numerous foci of inflammatory cells was seen in the livers of aged mice exposed to moderate ethanol (Fig. 3A and B). No micro- or macro-vesicular steatosis was observed in either ethanol group regardless of age, indicating that, at these levels, and for this length of treatment, ethanol is not inducing lipid accumulation and steatosis, as is often observed with higher ethanol exposure paradigms [45]. Moreover, no elevation of liver enzymes (aspartate transaminase (AST) and alanine aminotransferase (ALT)) occurred (data not shown). Overall, these observations suggests an ethanol-dependent liver hyper-inflammatory state in aged mice following moderate ethanol exposure.

Analysis of hepatic pro-inflammatory cytokine gene expression revealed that expression of the pro-inflammatory cytokine interleukin-1 beta (*Il1b*) was upregulated 4.6-fold (*p* < 0.05) in liver of aged mice exposed to ethanol relative young mice not exposed the ethanol (Fig. 3C). A moderate, but not significant, elevation in baseline tumor necrosis factor α (*Tnf*) mRNA levels in livers from aged mice, regardless of treatment, was also seen (Fig. 3D). The heightened basal cytokine level with advanced age is in agreement with the well described rise in baseline levels of circulating TNFα in “inflamm-aging” [46]. Analysis of expression of pro-inflammatory chemokine genes revealed that ethanol exposure in aged mice induced a significant 3-fold (*p* < 0.005) upregulation of expression of the monocyte chemoattractant protein-1 (C-C Motif Chemokine Ligand 2) *Ccl2* compared to young vehicle mice (Fig. 3E). This was in contrast to livers from young mice in which ethanol did not alter hepatic *Ccl2* expression. There was also an age-specific upregulation in expression of the neutrophil chemokine (C-X-C Motif Chemokine Ligand 2) *Cxcl2* (Fig. 3F), although this was not further elevated in livers of ethanol exposed aged mice.

**Fig. 4:**
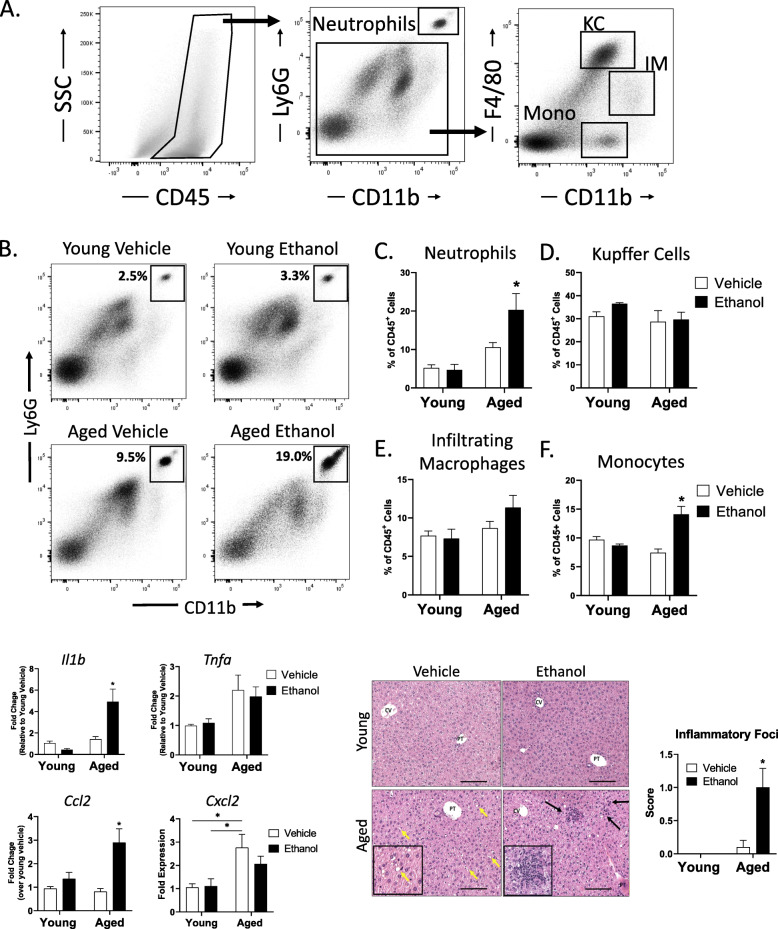
Moderate ethanol exposure in aged mice leads to increased neutrophils and monocytes in the liver.Flow cytometric analysis of hepatic non-parenchymal cells from aged and young mice given vehicle or ethanol. **A** Representative density plots showing gating strategy to define hepatic neutrophil, Kupffer Cell (KC), and infiltrating macrophage (IM) and monocyte populations. **B** Representative density plots showing the percent of hepatic CD45^+^ cells that are neutrophils. **C-F.** Bar charts showing the percent of CD45^+^ cells that are neutrophils, Kupffer cells, infiltrating macrophages and monocytes in the indicated treatment groups. *n* = 4–8 mice per group. Data shown are mean ± SEM. *p < 0.05 from all other groups by two-way ANOVA with Tukey’s multiple comparisons test

Finally, we performed flow cytometric analysis of non-parenchymal cells isolated from the livers of young and aged mice following vehicle or ethanol exposure to further characterize the myeloid cell phenotype based on expression of the surface markers CD11b, Ly6G and F4/80 (Fig. 4A). Levels of hepatic neutrophils, defined as the percent of CD45^+^ cells that are CD11b^+^Ly6G^+^, were enriched in the livers of aged mice exposed to ethanol compared to young ethanol treated mice with a mean of 22 % versus 8 % (p < 0.005) (Fig. 4B and C). Murine non-neutrophil hepatic myeloid cell populations can be further separated into monocytes, infiltrating macrophages (IM), and resident Kupffer cells by expression levels of the cell surface markers F4/80 and CD11b (Fig. 4A). Kupffer cells are defined as Ly6G^−^F4/80^hi^CD11b^low^, IM are Ly6G^−^F4/80^low^CD11b^hi^ and monocytes are Ly6G^−^ F4/80^−^CD11b^+^. Analysis of hepatic macrophages in our model showed that, while there was no significant change in the Kupffer cells or infiltrating macrophage population in aged mice (Fig. 4D and E), there was an ethanol-dependent increase in monocytes in the livers of aged mice given ethanol (Fig. 4F). This is further supported by the elevated CCL2 expression in the livers of aged mice exposed to ethanol (Fig. 3E). Overall, these data demonstrate that moderate ethanol exposure leads to hepatic inflammation, increased hepatic neutrophils and monocytes and elevated expression of pro-inflammatory chemokine genes in the livers of aged mice compared to young mice.

## Discussion

Chronic exposure to high levels of ethanol is known to have a number of detrimental effects on the gastrointestinal tract, causing intestinal barrier breakdown and significant hepatic inflammation (reviewed in [7]). However, little is known regarding the gastrointestinal effects of alcohol on older subjects, nor is it known whether more moderate levels of alcohol alter the gut and liver in a similar manner. In this study, we established a murine model of ethanol exposure in young and aged mice that reflects the moderate drinking patterns of a majority of the elderly, allowing us to characterize the intestinal and hepatic response to ethanol exposure in subjects of advanced age. Interestingly, we found that multi-day exposure to moderate levels of ethanol over a 4 week period, led to impaired intestinal barrier function in aged, but not young mice. iFABP is highly expressed in the intestinal epithelial cells of the small and large intestine and leakage of this protein into the circulation occurs following intestinal damage. We observed elevated serum levels of iFABP only in aged mice given ethanol (Fig. 1B). Ethanol exposure in aged mice also promoted leakage of FITC-dextran into the blood (Fig. 1C). We have observed an increase in serum FITC-dextran in aged mice as early as 3 days after ethanol exposure (McMahan, RM and Kovacs EJ, unpublished observation), well before there is evidence of liver inflammation and damage, suggesting that changes in intestinal permeability occur prior to liver inflammation. However, moderate levels of ethanol in aged mice also resulted in marked accumulation of bacteria in the MLN (Fig. 1A) and while more bacterial content in MLN could result from heightened intestinal barrier dysfunction, it could also reflect dysfunction within the intestinal or lymphatic phagocyte populations [38]. Future studies investigating the effect of age on intestinal macrophage populations, including their phenotype and function after ethanol exposure are warranted.

Direct intragastric administration of high concentrations of ethanol can lead to histological changes in the gastric mucosa in young rats [47]. In our model there were no gross alterations in intestinal epithelial barrier architecture with age or ethanol that could explain the decreased barrier function. There was a moderate, but non-significant, increase in baseline transcription levels of *Reg3g* in the ileum of aged mice, which has been observed in other models of aged mice [48]. This is similar to what is seen with increased baseline inflammatory cytokine levels in “inflamm-aging” and may represent a compensatory mechanism in the aging gut [46]. However, in young mice, multi-day moderate ethanol induced a more substantial upregulation of genes encoding intestinal AMPs (*Reg3g, Reg3b*), while in aged mice, there is a distinct lack of upregulation of these protective factors. Reg3g and Reg3b are secreted C-type lectins with strong bactericidal activity, forming a chemical barrier between the luminal surface of the gut and the commensal bacteria [9]. These AMPs play an important role in the maintenance of intestinal barrier integrity, as impaired intestinal expression leads to bacterial translocation to the MLN and liver [17]. Others have shown, in murine models of chronic ethanol exposure that employ much higher levels of ethanol (> 3 g/kg), that there is a downregulation of *Reg3g* and *Reg3b* in the ileum of young mice [14]. This is divergent from the upregulation that we observed in our more moderate ethanol exposure regimen suggesting the intestinal Reg3 response to ethanol is be dose-dependent. It is of interest that the expression of the tight junction protein, *Cldn1* was also upregulated in response to moderate ethanol in young, but not aged, mice. Claudins can be categorized as barrier-forming or pore-forming depending on their effect on epithelial cell function [49], and Claudin-1 is an important barrier-forming, or “tightening” claudin [41]. Collectively, these data would suggest that in young mice, moderate levels of ethanol lead to upregulation of barrier protective factors, such as AMPs and claudins, and this response is absent in the ileum of aged mice, further demonstrating the importance of studying the effects of moderate levels of ethanol in the intestine.

In addition to the altered intestinal parameters, aged mice also demonstrated significant alcohol-induced liver inflammation compared to young mice, with increased inflammatory foci and IL-1β mRNA expression after 4 weeks of multi-day ethanol exposure. The hepatic inflammatory infiltrate in aged mice given ethanol was primarily neutrophils and monocytes suggesting this model represents early stages of liver inflammation. The increase in hepatic monocytes and *Ccl2* in the absence of increased infiltrating macrophages in aged mice given ethanol also supports this as the liver are in the early stages of recruiting pro-inflammatory monocytes. While expression of *Cxcl2* was not increased in the livers of our mice at this time point, it may be that the expression neutrophil chemokines happens earlier post-ethanol exposure as seen in other liver inflammation models [50].

While these experiments were not designed to address the source of hepatic inflammation in this model, it is likely that impaired integrity of the intestinal epithelial barrier and MAMP exposure in the aged liver is contributing to the phenotype. IL-1β activation is triggered by various danger signals, including MAMPs, and is an important player in a number of inflammatory liver diseases [51]. However, production of reactive oxygen species during the metabolism of ethanol by cytochrome P450 2E1 (CYP2E1) can also contribute to alcohol-induced liver damage [52]. Furthermore, it has been shown that middle aged mice are more susceptible to liver inflammation induced by exposure to high concentrations of chronic plus binge ethanol due to altered expression of the sirtuin SIRT1 [53]. Therefore, we cannot rule out the possibility that the liver inflammation we observe in the livers of aged mice in our model results from a heightened susceptibility to ethanol-induced damage. Future experiments to determine if improving the intestinal barrier in aged mice eliminates the increased hepatic inflammation in response to ethanol would help clarify this mechanism [32].

## Conclusions

In conclusion, we have established a novel murine model that reflects a more clinically relevant ethanol consumption pattern in aged subjects and demonstrating for the first time that that aged mice are more vulnerable to the effects of moderate ethanol exposure. These finding have important implications for current recommended alcohol intake levels in patients over 65 years, as this model utilized alcohol levels just at, or even lower than, the recommended consumption of no more than 2 drinks per day or 7 drinks per week [54]. Although the liver inflammation our moderate ethanol study was less dramatic than what is seen in other more severe models of alcoholic liver disease, utilizing very high levels of ethanol, in aged mice, the more moderate ethanol exposure increased multiple inflammatory parameters, including a 4.6-fold increase in hepatic *Il1b* expression (Fig. 3C) along with a 7.5-fold increase in hepatic neutrophils (Fig. 4C). Importantly, this baseline inflammation could render the liver of aged subjects more susceptible to a “second hit,” lowering tolerance to other known liver stressors, such as acetaminophen [55], bile acids [56] or consumption of high fat or high sucrose diets [57]. In addition, it is possible that longer exposure to moderate ethanol in aged mice could lead to a more severe decline in liver function. The present model also demonstrates a clear age-related breakdown of the intestinal barrier following moderate ethanol consumption that could have implications for a number of conditions and diseases associated with age including obesity, cardiovascular disease and neurodegenerative diseases [58]. Taken together, this data provides critical insight into the impact of age on the intestinal and hepatic response to alcohol and highlights pathways for future exploration and clinical intervention.

## Data Availability

Data sharing not applicable to this article as no datasets were generated or analyzed during the current study.
