# Correction to: A novel murine model of multi-day moderate ethanol exposure reveals increased intestinal dysfunction and liver inflammation with age

**DOI:** 10.1186/s12979-021-00250-z

**Published:** 2021-10-21

**Authors:** Rachel H. McMahan, Kevin M. Najarro, Juliet E. Mullen, Madison T. Paul, David J. Orlicky, Holly J. Hulsebus, Elizabeth J. Kovacs

**Affiliations:** 1 Department of Surgery, Division of GI, Trauma and Endocrine Surgery, and Alcohol Research Program, University of Colorado Denver, Anschutz Medical Campus, 12,700 East 19th Ave, RC2, Mail Stop #8620, Aurora, CO 80045 USA; 2 GI and Liver Innate Immune Program, University of Colorado Denver, Anschutz Medical Campus, Aurora, CO 80045 USA; 3 Department of Pathology, University of Colorado Denver, Anschutz Medical Campus, Aurora, CO 80045 USA; 4 Department of Immunology and Microbiology, University of Colorado Denver, Anschutz Medical Campus, Aurora, CO 80045 USA

**Correction to: Immun Ageing 18, 37 (2021)**

**https://doi.org/10.1186/s12979-021-00247-8**

Following publication of the original article [1], the authors reported a publishing error in which images from Fig. 3 were inserted into Fig. 4 (the last 6 images). The correct Fig. 4 is presented below.

**Fig. 4:**
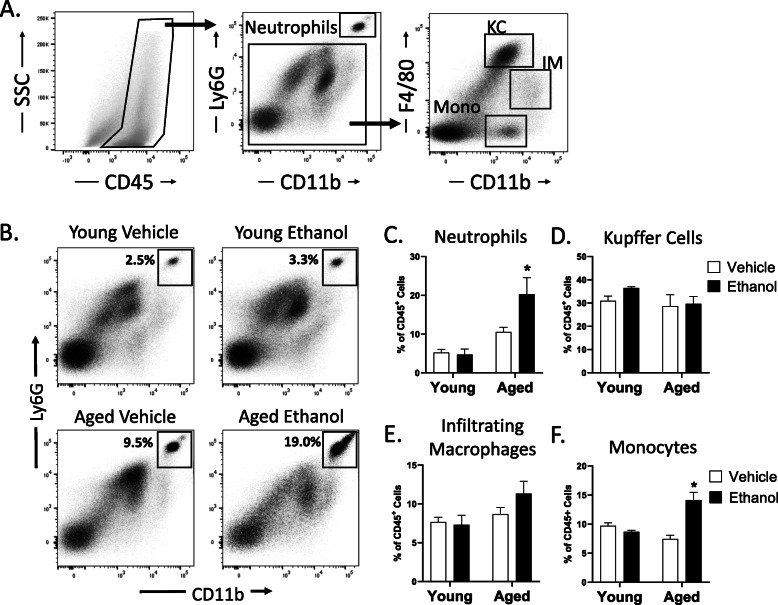
Moderate ethanol exposure in aged mice leads to increased neutrophils and monocytes in the liver. Flow cytometric analysis of hepatic non-parenchymal cells from aged and young mice given vehicle or ethanol. **A** Representative density plots showing gating strategy to define hepatic neutrophil, Kupffer Cell (KC), and infiltrating macrophage (IM) and monocyte populations. **B** Representative density plots showing the percent of hepatic CD45^+^ cells that are neutrophils. **C-F** Bar charts showing the percent of CD45^+^ cells that are neutrophils, Kupffer cells, infiltrating macrophages and monocytes in the indicated treatment groups. *n* = 4–8 mice per group. Data shown are mean ± SEM. ^*^p < 0.05 from all other groups by two-way ANOVA with Tukey’s multiple comparisons test

The original article [1] has been updated.
